# Knowledge and attitudes in the prevention of vertical transmission of HIV in referral hospitals

**DOI:** 10.4102/sajhivmed.v25i1.1553

**Published:** 2024-06-11

**Authors:** Patience D. Magugu, Melissa A. Lawler, Kimesh L. Naidoo

**Affiliations:** 1 Department of Paediatrics and Child Health, School of Clinical Medicine, University of KwaZulu-Natal, Durban, South Africa; 2 Prince Mshyeni Memorial Hospital, Durban, South Africa; 3 King Edward VIII Hospital, Durban, South Africa

**Keywords:** HIV, paediatrics, obstetrics, prevention of HIV vertical transmission programmes, PMTCT

## Abstract

**Background:**

Prevention of HIV vertical transmission programmes (VTPs) in South Africa has decreased paediatric HIV. These programmes require integration in referral hospitals.

**Objectives:**

To determine knowledge of and attitudes to the national VTP guidelines in staff from Obstetric and Paediatric disciplines at two referral hospitals.

**Method:**

Using a cross-sectional design, a questionnaire to assess knowledge of the guidelines and attitudes (awareness, ease-of-use and non-silo practice, measuring integrated practice) was developed and validated locally. Using standard statistical analyses, data from these questionnaires were used to draw comparisons and determine factors associated with knowledge and attitudes.

**Results:**

Of the 249 participants, 138 (55.4%) were in obstetrics, 125 (50.2%) were nurses, and 168 (67.5%) self-identified as junior staff. Knowledge scores were good, median score (Q1–Q3) was 91.7% (79.1–95.8), and higher in those who had discipline-specific training (*P* = 0.003). Junior staff (*P* = 0.002) had higher knowledge levels than senior staff. Most (80%) found the guidelines easy to use and had good awareness, which correlated with knowledge and training. Gaps included understanding of antenatal testing of HIV-negative women and timelines for neonatal HIV testing. Staff scored poorly on integrated practice; the median score (Q1–Q3) was 50% (33.3–58.3), which was inversely correlated with knowledge (*r*= –0.146, *n* = 249, *P* = 0.022).

**Conclusion:**

Staff in referral hospitals appear to be practising within silos when implementing VTPs, and this may result in failures to ensure integrated practice. Regularised interdisciplinary and interprofessional training may be important to ensure the integrated implementation of VTPs in referral hospitals.

**What this study adds:** Interdisciplinary and interprofessional training in VTP in referral hospitals needs to focus on key areas to ensure integration of practice.

## Introduction

Prevention of HIV vertical transmission programmes (VTP) in South Africa (SA) have decreased the HIV burden in paediatrics significantly by reducing transmission from 9.6% in 2008 to 0.9% in 2016.^1,2^ This decrease in HIV transmission is largely due to implementation, at scale, of evidence-based guidelines together with the provision of resources for HIV and viral load (VL) testing as well as an uninterrupted supply of appropriate antiretroviral drugs.^1^ The national HIV guidelines went through multiple iterations since the introduction of antiretroviral therapy in 2004, especially with regard to HIV testing, monitoring (CD4 count and VL) and the antiretroviral drug regimens used for both mothers and newborns.^3,4^ With these frequent changes in complex interventions, regular training of healthcare workers to ensure adequate knowledge and to instil supportive attitudes has been recognised as an essential component of successful implementation in VTP.^1,5^

Currently, SA has not achieved the 90-90-90 targets set by the World Health Organization (WHO), nor has it satisfied the criteria for elimination of vertical transmission of HIV, which requires a less than 0.05% transmission rate (based on HIV birth testing) to be achieved.^6,7^ Gaps in implementing VTPs need to be identified to achieve these targets. Poor knowledge and attitudes amongst healthcare workers, rising incident HIV infections, and poorly coordinated and integrated services have been identified as possible challenges that have resulted in SA failing to eliminate vertical transmission of HIV.^5,8^

The VTP programmes, both in design and implementation, have adopted a purposive primary healthcare approach focusing on midwife-run obstetric units (MOUs) and clinics where most low-risk pregnant women should be delivering their babies and then returning with their newborns for follow-up.^9,10^ Despite the South African government’s efforts to decentralise healthcare services in accordance with this primary care model, a significant number of women who ought to deliver in primary healthcare settings are still delivering in referral hospitals.^11^ These are hospitals based in each health region and serve as referral hospitals for complex, high-risk patients. These hospitals have discipline-trained staff (both nursing and medical) working solely within each discipline. In urban metropolitan centres in SA, large numbers of women categorised as having low-risk pregnancies as well as high-risk pregnancies are delivered in these referral hospitals.^12^

The implementation of an integrated VTP programme with multiple components spanning antenatal, peripartum, postnatal, and neonatal periods, requires coordination and cooperation.^10^ Whilst evaluation of VTP programmes has included referral hospitals, most have focused on the district health system, specifically at the primary healthcare level.^13^ National health priority programmes like the VTP programme are introduced into a fragmented healthcare system and this has been noted as a major obstacle to providing a continuum of care.^14^

Inadequate knowledge of VTP programmes among healthcare workers has been documented as a factor that influences the success of VTP.^13,15,16^ Individual factors have been identified as affecting healthcare workers’ attitudes to HIV prevention programmes, including the age of the healthcare worker, experience in managing HIV, gender, and occupation.^5^ It is not known whether attitudes amongst healthcare workers towards the VTP programmes vary with the types of training received.

This study evaluates the knowledge and attitudes on core aspects of the national VTP programme amongst nurses and medical doctors within specialist disciplines in referral hospitals situated in an HIV-endemic area. The study also compares these findings between obstetric and paediatric staff to identify gaps in care and to determine solutions to ensure the elimination of vertical HIV transmission.

## Research methods and design

### Study design

We conducted a cross-sectional descriptive study over 9 months, from 01 September 2022 to 31 May 2023. This sampling period was chosen to include multiple groups of newly qualified doctors (interns) and junior nurses who work in these units.

### Setting

The study was conducted in the obstetric and paediatric departments of two regional referral hospitals in Durban, KwaZulu-Natal. All staff in these disciplines are expected to attend and complete discipline-specific training on the national VTP. Combined, both these hospitals delivered a monthly average of 1600 newborns over the study period, including babies of mothers referred for high-risk obstetric and/or neonatal care from MOUs, primary healthcare clinics, and district hospitals.^17^ The documented HIV antenatal seroprevalence of mothers in the geographical area served by these hospitals is amongst the highest in the world at 40.9% (95% confidence interval [CI]: 39.6% – 42.3%).^18^

### Study population and sampling strategy

Every medical doctor and nurse in all the departments was approached to participate in the study. This included both those working at day and at night, and all levels of staff, from interns to consultants and enrolled nurses to matrons in charge. These participants were categorised for the purposes of this study as junior, if they had five or fewer years of experience, and senior, if they had more than five years in their respective discipline.

### Data collection

A questionnaire was developed to obtain data to assess both knowledge and attitude (Online Appendix 1 includes the final survey instrument and the scoring rubric). To assess knowledge, the three investigators consulted the national consolidated guidelines on VTP to identify key knowledge areas that all staff managing pregnant women or their newborns should have been trained on prior to commencing practice.^10^ Questions related to key knowledge were developed and circulated for input from an expert panel of medical and nursing managers of VTP programmes in both hospitals. In addition, a series of questions on determining attitudes and scoring rubrics was also developed and similarly circulated. After multiple iterations of a consultative face-to-face process with members of the expert panel, electronic consensus was obtained on the questions to be included in questionnaire. The three key areas to assess knowledge were antenatal care (assessing knowledge on maternal VL testing), labour ward practices (assessing knowledge on maternal HIV risk categorisation), and postnatal care (assessing knowledge on both HIV birth testing and infant prophylaxis). The three key areas identified to assess attitude included awareness (determined by evaluating the urgency of action required with HIV-exposed infants requiring testing and treating), attitude to the usability of both maternal and neonatal guidelines (measured by determining perceptions on the ‘ease-of-use’ of these guidelines), and attitude to integrated care (measured with questions about which healthcare worker is primarily accountable; this was termed ‘perceptions of “non-silo practice”’ for the purposes of this study).

The scoring rubric developed allowed for a determination of levels of knowledge with answers differentially scored. This was purposely done to determine participants’ levels of knowledge using answers based on the current VTP guidelines as the optimal level for scoring, incorrect answers based on information from outdated guidelines as the intermediate score, and all other answers or indicating having no knowledge as the lowest score. The questionnaire, including the scoring rubric developed, was piloted with groups of junior doctors and nurses three months before the study period to ensure face validity. Various questions were reviewed, and the scoring rubric adjusted, and once a consensus with changes was reached with all investigators, the questionnaire and scoring rubric were finalised.

In order to determine attitudes related to the integration of care, the questions on non-silo practice were specifically developed to determine how participants viewed the responsibility of care to each component of the VTP programme. Poorly integrated care was viewed as ascribing responsibility to only a specific individual whilst integrated care (non-silo care) was considered when participants recognised that all members of the health team were equally responsible for that aspect of care. Thus, total non-silo practice scores were considered as a surrogate score to indicate the likelihood of integrated implementation of VTP guidelines.^19^ The questionnaire also obtained demographic information on age, self-identified gender, years of experience, and exposure to formal VTP training.

### Data analysis

The primary investigator verified and entered all data from the completed surveys into an Excel spreadsheet. Statistical analysis was performed using the R Core Team’s R Statistical computing software, version 3.6.3 (R Foundation for Statistical Computing, Vienna, Austria). The categorical variables were described as counts and percentage frequencies. Comparative analyses determined relationships between demographic characteristics, total and sub-component knowledge, awareness, ease-of-use, and non-silo practice scores. The Chi-square or Fisher’s exact test (small frequencies) was used to determine the association between categorical data. Logistic regression was further applied to determine knowledge relationships with attitude (awareness, usability and accountability) using odds ratios (OR), including 95% confidence intervals. All the inferential statistical analysis tests were conducted at 5% significance levels (*P* < 0.05).

### Ethical considerations

Ethical clearance to conduct this study was approved by the University of KwaZulu-Natal Biomedical Research Ethics Committee (BREC/00003828/2022), the KwaZulu-Natal Department of Health’s Provincial Health Research Ethics Committee, King Edward VIII and Prince Mshyeni Memorial Hospitals. All participants provided written consent for the study.

## Results

Of the 249 participants, 125 (50.2%) were nurses, and 124 (49.8%) were medical doctors. The response rate was 67.8% based on the hospital records of approximately 220 nurses and 147 medical doctors within these disciplines across the study period. The median age was 30 (Q1–Q3: 26–40) years, and most (*n* = 183; 73.5%) identified as female. Of all participants, 138 (55.4%) indicated that they worked in Obstetric disciplines, and 111 (44.6%) in Paediatric disciplines. Most self-identified as being junior, with 124 (49.8%) having < 5 years’ experience in their discipline, 52 (20.9%) between 5 and 10 years, 35 (14.1%) between 10 and 15 years, and 38 (15.3%) > 15 years. Of all the participants, 120 (48.2%) indicated they had received formal training in VTP implementation, 102 (41.0%) had received informal training (‘on-the-job’ training), and only 27 (10.8%) had received no training. Table 1 compares the demographic characteristics of participants in the Obstetric and Paediatric disciplines.

**TABLE 1:** Comparison of the demographic characteristics of participants between Obstetrics and Paediatric disciplines.

Demographic characteristics	Obstetrics (*n* = 138)					Paediatrics (*n* = 111)					*P*	Overall (*N* = 249)				
	Median	Q1–Q3	Min–Max	*n*	%	Median	Q1–Q3	Min–Max	*n*	%		Median	Q1–Q3	Min–Max	*n*	%
**Age (years)**	29	25–39	23–63	-	-	33	27–42	22–60	-	-	0.077	30	26–40	22–63	-	-
**Years qualified (years)**	-	-	-	-	-	-	-	-	-	-	0.109	-	-	-	-	-
< 5	-	-	-	74	53.6	-	-	-	50	45.0	-	-	-	-	124	49.8
5–10	-	-	-	32	23.2	-	-	-	20	18.0	-	-	-	-	52	20.9
10–15	-	-	-	14	10.1	-	-	-	21	18.9	-	-	-	-	35	14.1
> 15	-	-	-	18	13.0	-	-	-	20	18.0	-	-	-	-	38	15.3
**Gender**	-	-	-	-	-	-	-	-	-	-	0.903	-	-	-	-	-
Female	-	-	-	101	73.2	-	-	-	82	73.9	-	-	-	-	183	73.5
Male	-	-	-	37	26.8	-	-	-	29	26.1	-	-	-	-	66	26.5
**Profession**	-	-	-	-	-	-	-	-	-	-	0.275	-	-	-	-	-
Nurse	-	-	-	65	47.1	-	-	-	60	54.1	-	-	-	-	125	50.2
Doctor	-	-	-	73	52.9	-	-	-	51	45.9	-	-	-	-	124	49.8
**Level of staff in each profession**	-	-	-	-	-	-	-	-	-	-	0.013	-	-	-	-	-
Junior nurse	-	-	-	34	24.6	-	-	-	35	31.5	1.000	-	-	-	69	27.7
Junior doctor	-	-	-	65	47.1	-	-	-	34	30.6	0.037	-	-	-	99	39.8
Senior nurse	-	-	-	31	22.5	-	-	-	25	22.5	1.000	-	-	-	56	22.5
Senior doctor	-	-	-	8	5.8	-	-	-	17	15.3	0.074	-	-	-	25	10.0
**Training in VTP**	-	-	-	-	-	-	-	-	-	-	0.084	-	-	-	-	-
Formal training	-	-	-	74	53.6	-	-	-	46	41.4	-	-	-	-	120	48.2
Informal training	-	-	-	48	34.8	-	-	-	54	48.6	-	-	-	-	102	41.0
No training	-	-	-	16	11.6	-	-	-	11	9.9	-	-	-	-	27	10.8

VTP, prevention of vertical transmission of HIV.

### Knowledge of the VTP programme

Table 2 provides an overview of the knowledge scores and responses to individual questions and compares these responses between the Obstetric and Paediatric disciplines. Overall knowledge levels were high, with the median (Q1–Q3) total knowledge score at 91.7% (79.1–95.8; *P* = 0.007) for all participants.

**TABLE 2:** Knowledge scores and percentage responses to component questions of participants in Obstetrics and Paediatric specialities.

Component and sub-component questions	Obstetrics (*n* = 138)					Paediatrics (*n* = 111)					*P*	Overall (*N* = 249)				
	Median	Q1–Q3	Min–Max	*n*	%	Median	Q1–Q3	Min–Max	*n*	%		Median	Q1–Q3	Min–Max	*n*	%
Total knowledge score (%)	91.7	83.3–95.8	45.8–100.0	-	-	87.5	79.1–91.7	20.8–100.0	-	-	0.007	91.7	79.1–95.8	-	-	-
**Antenatal knowledge score (%)**	83.3	83.3–100.0	33.3–100.0	-	-	83.3	66.7–100.0	0.0–100.0	-	-	0.277	83.3	83.3–100.0	0.0–100.0	-	-
**Antenatal knowledge score component questions** † ‡																
**Optimal maternal viral load in HIV-positive mothers on HAART**	-	-	-	-	-	-	-	-	-	-	0.740	-	-	-	-	-
< 1000† (new guidelines < 50)	-	-	-	127	92.0	-	-	-	99	89.2	-	-	-	-	226	90.8
1000+	-	-	-	8	5.8	-	-	-	8	7.2	-	-	-	-	16	6.4
Not sure	-	-	-	3	2.2	-	-	-	4	3.6	-	-	-	-	7	2.8
**Optimal VL testing frequency in HIV-positive mothers**	-	-	-			-	-	-			0.126	-	-	-	-	-
Depends on maternal viral load†	-	-	-	112	81.2	-	-	-	84	75.7	-	-	-	-	196	78.7
Every antenatal care visit	-	-	-	23	16.7	-	-	-	17	15.3	-	-	-	-	40	16.1
Monthly	-	-	-	2	1.4	-	-	-	6	5.4	-	-	-	-	8	3.2
Not sure	-	-	-	1	0.7	-	-	-	4	3.6	-	-	-	-	5	2.0
**Optimal process to do HIV test if mother HIV-negative in antenatal care**	-	-	-			-	-	-			0.470	-	-	-	-	-
Mother must request	-	-	-	59	44.0	-	-	-	46	41.8	-	-	-	-	105	43.0
Encouraged at every basic antenatal care (BANC) visit†	-	-	-	69	51.5	-	-	-	57	51.8	-	-	-	-	126	51.6
Mandatory monthly test	-	-	-	1	0.7	-	-	-	4	3.6	-	-	-	-	5	2.0
Not sure	-	-	-	5	3.7	-	-	-	3	2.7	-	-	-	-	8	3.3
**Labour ward knowledge score (%)**	100.0	83.3–100.0	50.0–100.0	-	-	100.0	83.3–100.0	16.7–100.0	-	-	0.235	100.0	83.3–100.0	16.7–100.0	-	-
**Labour ward knowledge score component questions** † ‡																
**Need to repeat VL at delivery in all mothers** ‡	-	-	-	-	-	-	-	-	-	-	0.254	-	-	-	-	-
No	-	-	-	9	6.5	-	-	-	8	7.2	-	-	-	-	17	6.8
Yes†	-	-	-	103	74.6	-	-	-	71	64.0	-	-	-	-	174	69.9
Depends on mother’s last viral load	-	-	-	25	18.1	-	-	-	30	27.0	-	-	-	-	55	22.1
Not sure	-	-	-	1	0.7	-	-	-	2	1.8	-	-	-	-	3	1.2
**HIV risk in a mother without a VL in Labour** ‡	-	-	-			-	-	-			0.287	-	-	-	-	-
Low	-	-	-	4	2.9	-	-	-	1	0.9	-	-	-	-	5	2.0
Intermediate	-	-	-	2	1.4	-	-	-	3	2.7	-	-	-	-	5	2.0
High†	-	-	-	132	95.7	-	-	-	105	94.6	-	-	-	-	237	95.2
Not sure	-	-	-	0	0.0	-	-	-	2	1.8	-	-	-	-	2	0.8
**HIV risk if newly diagnosed HIV-positive in Labour** ‡	-	-	-	-	-	-	-	-	-	-	0.549	-	-	-	-	-
Low	-	-	-	0	0.0	-	-	-	2	1.8	-	-	-	-	2	0.8
Intermediate	-	-	-	2	1.4	-	-	-	1	0.9	-	-	-	-	3	1.2
High†	-	-	-	135	97.8	-	-	-	107	96.4	-	-	-	-	242	97.2
Not sure	-	-	-	1	0.7	-	-	-	1	0.9	-	-	-	-	2	0.8
**Infant testing knowledge score (%)**	83.3	83.3–100.0	33.3–100.0			83.3	66.7–100.0		0.0–100.0		< 0.001	83.3	66.7–100.0	0.0–100.0	-	-
**Infant testing knowledge score component questions** † ‡																
**Optimal time to do a DNA PCR test on the newborn of HIV-positive mum** † ‡											0.004	-	-	-	-	-
Immediately after birth (< 24 h)	-	-	-	127	92.0	-	-	-	97	87.4	-	-	-	-	224	90.0
Within 24–48 h of birth†	-	-	-	9	6.5	-	-	-	3	2.7	-	-	-	-	12	4.8
Within 48–72 h of birth	-	-	-	0	0.0	-	-	-	7	6.3	-	-	-	-	7	2.8
Not sure	-	-	-	2	1.4	-	-	-	4	3.6	-	-	-	-	6	2.4
**Minimum waiting time for check DNA PCR test on newborn baby test** ‡											< 0.001	-	-	-	-	-
Within 48 h	-	-	-	29	21.0	-	-	-	45	40.5	-	-	-	-	74	29.7
Within 7 days†	-	-	-	95	68.8	-	-	-	50	45.0	-	-	-	-	145	58.2
Within 1 month	-	-	-	13	9.4	-	-	-	12	10.8	-	-	-	-	25	10.0
Not sure	-	-	-	1	0.7	-	-	-	4	3.6	-	-	-	-	5	2.0
**Waiting time baby test post breastfeeding** ‡											< 0.001	-	-	-	-	-
7 days	-	-	-	4	2.9	-	-	-	-	-	-	-	-	-	24	9.7
14 days	-	-	-	23	16.7	-	-	-	-	-	-	-	-	-	39	15.7
6 weeks†	-	-	-	104	75.4	-	-	-	-	-	-	-	-	-	172	69.4
Not sure	-	-	-	7	5.1	-	-	-	-	-	-	-	-	-	13	5.2
**Infant prophylaxis knowledge score (%)**	100.0	100.0–100.0	0.0–100.0	-	-	100.0	100.0–100.0	0.0–100.0	-	-	0.905	100.0	100.0–100.0	0.0–100.0	-	-
**Infant prophylaxis knowledge score component questions** † ‡																
**Standard low-risk infant prophylaxis** ‡											0.503	-	-	-	-	-
Nevirapine†	-	-	-	123	89.1	-	-	-	99	89.2	-	-	-	-	222	89.2
Nevirapine and Zidovudine	-	-	-	12	8.7	-	-	-	7	6.3	-	-	-	-	19	7.6
Zidovudine only	-	-	-	3	2.2	-	-	-	5	4.5	-	-	-	-	8	3.2
**Infant prophylaxis changes if maternal VL is high** ‡											0.436	-	-	-	-	-
Add Zidovudine to Nevirapine†	-	-	-	124	89.9	-	-	-	99	89.2	-	-	-	-	223	89.6
Longer duration of Nevirapine	-	-	-	5	3.6	-	-	-	7	6.3	-	-	-	-	12	4.8
It stays the same	-	-	-	5	3.6	-	-	-	1	0.9	-	-	-	-	6	2.4
Not sure	-	-	-	4	2.9	-	-	-	4	3.6	-	-	-	-	8	3.2
**Infant prophylaxis changes if maternal VL is undetectable** ‡											0.043	-	-	-	-	-
Nevirapine only†	-	-	-	109	79.0	-	-	-	86	77.5	-	-	-	-	195	78.3
Stop all antiretrovirals	-	-	-	8	5.8	-	-	-	5	4.5	-	-	-	-	13	5.2
Either Zidovudine or Nevirapine	-	-	-	21	15.2	-	-	-	14	12.6	-	-	-	-	35	14.1
Not sure	-	-	-	0	0.0	-	-	-	6	5.4	-	-	-	-	6	2.4

HAART, highly active antiretroviral therapy; VL, viral load; PCR, polymerase chain reaction.

† , expected answer.

‡ , Percentage (%) participants per answer.

Most participants (*n* = 226, 90.8%) had adequate knowledge of VL monitoring level cut-offs, and 196 (78.7%) had adequate knowledge of testing frequency. Only 126 (51.6%) indicated that HIV-negative mothers need to be encouraged to test at every basic antenatal care (BANC) visit, with 105 (43.0%) indicating that the mother should request this test. Regarding VL testing in labour, 174 (69.9%) indicated that all mothers require a repeat test, with 55 (22.1%) indicating that a previous VL result could determine this need. Most, participants (*n* = 237, 95.2%) recognised that a mother without a VL result in labour must be categorised as a high-risk patient and 242 (97.2%) recognised that a newly diagnosed HIV-positive mother must be categorised as high risk. Most participants (*n* = 224, 90.0%) indicated that birth HIV testing in newborns should be done immediately after delivery, and only 12 (4.8%) correctly indicated that this testing can be done within a 24 h – 48 h period.

For the infant testing knowledge, the median (Q1–Q3) score was 83.3% (66.7–100.0; *P* < 0.001); however, significant differences were identified between obstetric and paediatric staff regarding the optimal time to do DNA polymerase chain reaction (PCR) tests for newborns post-delivery and following cessation of breastfeeding. There were significant differences in the responses from those in paediatrics and obstetrics regarding the optimal waiting time for birth PCR results, with 40.5% (*n* = 45) of paediatric staff indicating that results should optimally be obtained within 48 h of birth.

Most participants had adequate knowledge of infant drug prophylaxis changes with low- and high-risk, and virally suppressed, mothers.

### Awareness and ease of protocol use

When assessing awareness of participants on the need for urgent action required, most had adequate knowledge regarding the fact that certain at-risk timepoints must be treated as emergencies, namely high-risk deliveries (*n* = 211, 84.7%), birth testing (*n* = 225, 90.4%) and newly diagnosed HIV-positive newborns (*n* = 241, 96.8%). Most participants (*n* = 211 [84.7%] for the maternal component, and *n* = 208 [83.5%] for the infant component) indicated that the VTP guidelines were easy to understand and use. Table 3 indicates the awareness and ease-of-use scores, as well as responses to component questions.

**TABLE 3:** Comparisons of attitudes between obstetrics and paediatrics discipline health personnel.

Component and sub-component questions	Obstetrics (*n* = 138)					Paediatrics (*n* = 111)					*P*	Overall (*N* = 249)				
	Median	Q1–Q3	Min–Max	*n*	%	Median	Q1–Q3	Min–Max	*n*	%		Median	Q1–Q3	Min–Max	*n*	%
**Attitude (Awareness and Ease of VTP guideline use)**																
**Awareness scores (%)**	83.3	75.0–91.7.0	41.7–100.0	-	-	83	79.2–100.0	50.0–100.0	-	-	0.455	83.3	75.0–91.7	50.0–100.0	-	-
**Awareness component questions** † ‡																
**High-risk HIV delivery**	-	-	-	-	-	-	-	-	-	-	0.278	-	-	-	-	-
Does not require urgent action	-	-	-	18	13.0	-	-	-	20	18.0		-	-	-	38	15.3
Requires urgent action†	-	-	-	120	87.0	-	-	-	91	82.0		-	-	-	211	84.7
**Infant HIV test post-delivery**	-	-	-			-	-	-			0.463	-	-	-		
Requires urgent action†	-	-	-	123	89.1	-	-	-	102	91.9		-	-	-	225	90.4
Does not require urgent action	-	-	-	15	10.9	-	-	-	9	8.1		-	-	-	24	9.6
**Newly diagnosed positive infant**	-	-	-	-	-	-	-	-	-	-	0.735	-	-	-	-	-
Does not require urgent action	-	-	-	5	3.6	-	-	-	3	2.7		-	-	-	8	3.2
Requires urgent action†	-	-	-	133	96.4	-	-	-	108	97.3		-	-	-	241	96.8
**Protocol ease-of-use score (%)**	80.0	80.0–100.0	40.0–100.0	-	-	80.0	80.0–100.0	20.0–100.0	-	-	0.870	80.0	80.0–100.0	20.0–100.0	-	-
**Ease-of-use component questions** ‡																
**Maternal protocol for VTP** ‡							-	-	-	-	0.983	-	-	-	-	-
Not easy to use	-	-	-	21	15.2	-	-	-	17	15.3	-	-	-	-	38.0	15.3
Easy to use	-	-	-	117	84.8	-	-	-	94	84.7	-	-	-	-	211.0	84.7
**Infants protocol for VTP** ‡							-	-	-		0.434	-	-	-	-	-
Not easy to use	-	-	-	25	18.1	-	-	-	16	14.4	-	-	-	-	41	16.5
Easy to use	-	-	-	113	81.9	-	-	-	95	85.6	-	-	-	-	208	83.5

VTP, prevention of vertical transmission of HIV.

† , expected answer.

‡ , Percentage (%) participants per answer.

### Assessing non-silo practice

The median (Q1–Q3) non-silo practice scores were only 50.0% (33.3–58.3; *P* = 0.355) for all participants, and there were no differences between staff in Obstetrics and Paediatrics regarding these responses. Only 22.5% (*n* = 56) of all participants indicated that all health personnel are responsible for antenatal HIV care in the VTP programme; instead, most (*n* = 193, 77.5%) indicated that only the midwives are responsible. Similarly, only 52 (20.9%) of participants believed all staff in the labour ward were responsible for implementing VTP, with 197 (79.1%) indicating that the labour ward doctors were solely responsible. In terms of infant testing for HIV and care of HIV infants, only 51 (20.5%) responded that all health personnel are jointly responsible, with 198 (79.5%) indicating that paediatric doctors are solely responsible. Table 4 indicates the non-silo practice scores of participants in Obstetrics and Paediatric specialities, and the responses per question.

**TABLE 4:** Non-silo practice scores of participants in Obstetrics and Paediatric specialities.

Component and sub-component questions	Obstetrics (*n* = 138)					Paediatrics (*n* = 111)					*P*	Overall (*N* = 249)				
	Median	(Q1–Q3)	(Min–Max)	*n*	%	Median	(Q1–Q3)	(Min–Max)	*n*	%		Median	(Q1–Q3)	(Min–Max)	*n*	%
**Non-silo practice scores (%)**	50	33.3–58.3	25.0–100.0	-	-	50	29.2–58.3	25.0–83.3	-	-	0.355	50	33.3–58.3	25.0–100.0	-	-
**Non-silo practice score component questions**																
**Antenatal clinic – VTP care** † ‡	-	-	-	-	-	-	-	-	-	-	0.991	-	-	-	-	-
All health jointly personnel responsible†	-	-	-	31	22.5	-	-	-	25	22.5	-	-	-	-	56	22.5
Midwife solely responsible	-	-	-	107	77.5	-	-	-	86	77.5	-	-	-	-	193	77.5
**Labour ward – VTP care** † ‡	-	-	-	-	-	-	-	-	-	-	0.797	-	-	-	-	-
All health personnel are jointly responsible†	-	-	-	28	20.3	-	-	-	24	21.6	-	-	-	-	52	20.9
Labour ward doctors are solely responsible	-	-	-	110	79.7	-	-	-	87	78.4	-	-	-	-	197	79.1
**Infant testing and care –VTP care** ‡	-	-	-	-	-	-	-	-	-	-	0.816	-	-	-	-	-
All health personnel are jointly responsible†	-	-	-	29	21.0	-	-	-	22	19.8	-	-	-	-	51	20.5
Paediatric doctors are solely responsible	-	-	-	109	79.0	-	-	-	89	80.2	-	-	-	-	198	79.5

VTP, prevention of vertical transmission of HIV.

† , expected answer.

‡ , Percentage (%) participants per answer.

### Professional category and knowledge and attitudes

Junior nurses and doctors had significantly higher median knowledge scores (Q1–Q3) (91.7% [83.3–95.8]) for total knowledge scores than senior nurses (87.5% [78.3–83.3]), and senior doctors (79.2% [70.8–91.7]). Table 5 lists the comparative scores between the various categories of staff. These differences were most significant concerning questions related to antenatal care (*P* < 0.001), infant testing (*P* < 0.001) and infant prophylaxis (*P* < 0.001). Significantly more junior nurses and doctors than senior nurses and doctors answered questions correctly regarding when to test pregnant women for HIV antenatally, when to test and what prophylaxis to give an HIV-exposed newborn.

**TABLE 5:** Comparison of knowledge, awareness and non-silo practice scores between all professional categories of health personnel.

Level	Junior nurses (*n* = 69)	Junior doctors (*n* = 99)	Senior nurses (*n* = 56)	Senior doctors (*n* = 25)	*P*	Overall (*N* = 249)
**Awareness score (%)**					< 0.001	-
Median	83.3	91.7	83.3	91.7	-	83.3
Q1–Q3	75.0–91.7	83.3–100.0	83.3–91.7	83.3–100.0	-	75.0–91.7
Min–Max	50.0–100.0	41.7–100.0	50.0–100.0	58.3–100.0	-	41.7–100.0
**Ease-of-use score (%)**					0.227	-
Median	80.0	80.0	80.0	80.0	-	80.0
Q1–Q3	80.0–90.0	80.0–100.0	80.0–100.0	80.0–90.0	-	80.0–100.0
Min–Max	40.0–100.0	40.0–100.0	20.0–100.0	30.0–100.0	-	20.0–100.0
**Non-silo practice score (%)**					0.043	-
Median	50.0	50.0	50.0	41.7	-	50.0
Q1–Q3	25.0–50.0	41.7–58.3	25.0–58.3	33.3–58.3	-	33.3–58.3
Min–Max	25.0–83.3	25.0–100.0	25.0–83.3	25.0–83.3	-	25.0–100.0
**ANC knowledge score (%)**					< 0.001	-
Median	100.0	83.3	83.3	83.3	-	83.3
Q1–Q3	83.3–100.0	83.3–83.3	33.3–83.3	41.7–83.3	-	83.3–100.0
Min–Max	33.3–100.0	50.0–100.0	33.3–100.0	0.0–100.0	-	8.3–100.0
**Labour ward knowledge score (%)**					0.030	-
Median	100.0	100.0	100.0	100.0	-	100.0
Q1–Q3	100.0–100.0	83.3–100.0	83.3–100.0	83.3–100.0	-	83.3–100.0
Min–Max	66.7–100.0	16.7–100.0	33.3–100.0	0.0–100.0	-	0.0–100.0
**Infant testing knowledge score (%)**					< 0.001	-
Median	83.3	100.0	66.7	100.0	-	83.3
Q1–Q3	66.7–100.0	83.3–100.0	66.7–100.0	83.3–100.0	-	66.7–100.0
Min–Max	8.3–100.0	8.3–100.0	8.3–100.0	25.0–100.0	-	8.3–100.0
**Infant prophylaxis knowledge score (%)**					< 0.001	-
Median	100.0	100.0	100.0	33.3	-	100.0
Q1–Q3	100.0–100.0	100.0–100.0	33.3–100.0	16.7–100.0	-	100.0–100.0
Min–Max	8.3–100.0	8.3–100.0	8.3–100.0	8.3–100.0	-	8.3–100.0
**Total knowledge score (%)**					0.002	-
Median	91.7	91.7	87.5	79.2	-	91.7
Q1–Q3	83.3–95.8	83.3–95.8	78.3–83.3	70.8–91.7	-	79.1–91.7
Min–Max	54.2–100.0	50.0–100.0	45.8–100.0	20.8–95.8	-	20.8–100.0

ANC, antenatal care.

Regarding awareness, there was also a significant difference (*P* < 0.001) between doctors (both junior and senior) and nurses (junior and senior) in recognising the need for urgent action required in high-risk deliveries, birth testing results and newly diagnosed HIV-positive newborns. More junior nurses and doctors than senior nurses and doctors indicated that the following clinical scenarios should be considered emergencies requiring urgent intervention: a women deemed high risk for HIV transmission in labour, HIV testing of a newborn of an HIV-positive mother, and providing antiretroviral treatment to a newly diagnosed HIV-positive newborn.

There were no significant differences between all categories of staff in ease-of-use (*P* = 0.227) and non-silo practice (*P* = 0.043) scores. However, junior doctors had higher mean non-silo practice scores, followed by senior doctors, senior nurses, and junior nurses.

**FIGURE 1:**
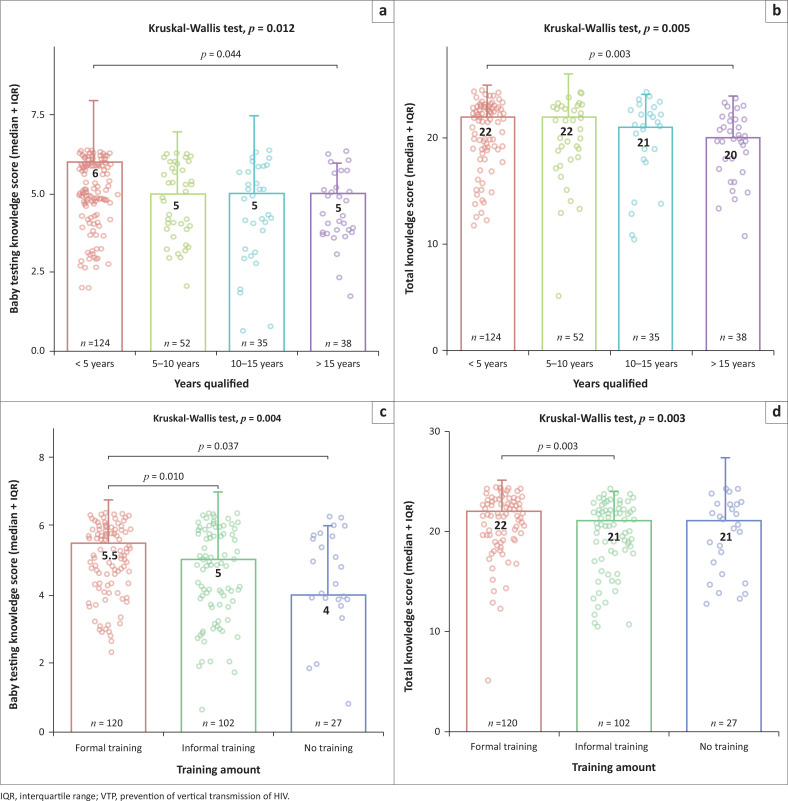
Comparison of knowledge scores, years qualified and formal training. (a) Comparisons of infant testing knowledge with years qualified (b) Comparisons of total knowledge scores with years qualified (c) Comparisons of infant testing knowledge with VTP training received (d) Comparisons of total knowledge scores with VTP training received.

The number of years of experience in a discipline was found to be important with those with less than five years of experience having a median (Q1–Q3) total knowledge score of 91.7% (83.3–95.8) compared with 83.3% (75.0–91.7; *P* = 0.005) in those with greater than 15 years’ experience. Participants who had less than 5 years’ experience answered more questions correctly on when to test a newborn baby for HIV than those participants with more than 15 years of experience. This was statistically significant and, considering all the questions on knowledge of key components of the VTP programme, a similar pattern was found with those with less than 5 years’ experience answering more questions correctly than those with more than 5 years’ experience. Figure 1 illustrates these findings, and Online Appendix 2 provides the detailed comparisons.

There were significant differences in total knowledge scores between those who indicated they had formal training in the VTP programme with median (Q1–Q3) scores of 91% (83.3–95.8; *P* = 0.004) compared with those with informal training and no training (87.5%; 68.8–95.8). Here, infant testing knowledge components highlighted the most significant differences in compared groups with differing VTP training. Figure 1 illustrates, and Online Appendix 3 provides, detailed comparisons.

### Associations between knowledge, non-silo practice, and ease-of-use scores

It was found that many participants with higher knowledge scores have lower non-silo scores (*r* = –0.146; *n* = 249; *P* = 0.022). It was also found that those participants with higher knowledge scores also had higher ease-of-use scores (*r* = 0.002; *n* = 249); however, this was not statistically significant.

## Discussion

This study provides data on knowledge levels and attitudes toward VTP from both Obstetric and Paediatric medical and nursing staff in two referral hospitals serving a population with high antenatal HIV seroprevalence rates. The participants in this study reflect the general demographic profile of staff responsible for implementing VTP guidelines across most SA hospitals: predominantly young, female, and designated junior within their respective departments.^1^ These staff should be targeted for formal training in VTP.^10^ In our study, 48.2%, just less than half of the participants, indicated having formal training. This corresponds with studies elsewhere earlier in the HIV epidemic and are of concern at this stage in SA’s response to the prevention of vertical transmission.^13,15^ Our study identifies that junior staff who rotate through different departments could be missing formal training if this is not regularised at orientation.

Our study identifies overall good knowledge levels across all participants, with a median score of 91.7%, which is higher than in previous studies in sub-Saharan Africa.^5,15,16^ As previously indicated, it is junior staff who are largely responsible for the implementation of the VTP programme, and this is reflected in the findings of our study which shows that junior staff have greater knowledge on key areas when compared with senior staff.^1^ This highlights a gap in the implementation process of VTP that is specific to referral hospitals. If senior staff have less practical knowledge of VTP, there is a concern that complicated cases requiring their input are being inadequately managed or management delegated, possibly to the fewer infectious disease sub-specialists. This is not sustainable, especially for the ‘at-scale’ need for implementation of VTP in HIV-burdened areas, where all generalist specialists are required to have adequate knowledge to support the ‘front-line’ junior staff with referred and complex cases.^20^

Despite the overall good knowledge scores, our study identifies gaps in specific components that are important in the attempt to eliminate vertical transmission of HIV.^1,20^ One identified gap in knowledge is the management of HIV-negative women in the antenatal clinic with regard to HIV testing. The failure to offer women an HIV test at every BANC attendance may fail to pick up newly acquired infections in HIV-negative women.^6,20^ In addition, participants in the study had inadequate knowledge of cut-off times for testing neonates, either post-delivery or upon cessation of breastfeeding. As vertical HIV transmission rates fall, the need to identify the fewer positive infants becomes crucial; hence, the need to comprehensively test HIV-exposed infants requires greater attention.^6,10,20^

In our study, we demonstrate a clear association between having formal training and having better knowledge levels, and this is corroborated in other studies.^10,15^ An additional aspect identified is the positive correlation between higher knowledge levels and recognising the need for urgent action that is required to be taken in specific clinical scenarios.

One of the key objectives of this study, which was purposively based across disciplines in a referral hospital, was to determine levels of integrated practice of a national priority health programme developed for primary healthcare implementation.^9,10^ This study corroborates major concerns previously expressed of a traditional siloed nature of healthcare provision persisting.^8,21,22^ The low non-silo practice scores, irrespective of professional category, discipline or years of experience, suggest that most staff in referral hospitals do not comprehend the need for integrated care, which is essential for the successful implementation of VTP programmes.^19,22,23^ This culture of working in silos and delegating certain healthcare workers as being responsible for each component of care exacerbates poor coordination between team members, especially required for the linkages between antenatal and labour ward care as well as the infant and maternal follow-up in the crucial post-partum period.^21^ Training on VTP in most referral hospitals is largely intra-professional and intra-disciplinary. Our study identified a significant negative correlation between knowledge scores and non-silo practice scores, suggesting that existing patterns of training may be contributing to the lack of integration and entrenching working in silos. We therefore strongly recommend that VTP training within referral hospitals be approached with an interprofessional and interdisciplinary ethos. Training needs to be included in interprofessional collaborative practice between obstetrics and paediatric teams, and this may improve care for these patients, specifically in referral hospitals, to demystify the issue of accountability and avoid losing patients to follow-up, especially for HIV-exposed infants and mothers living with HIV.^21^ Interprofessional collaborative practice in the setting of VTPs seems a necessary shift to improve outcomes for the mother–child dyad.^20,23^ These aspects may assist in closing those gaps as we hope to eliminate vertical transmission of HIV.

### Strengths and limitations

This comprehensive study assesses interprofessional knowledge and attitudes across all the components of the complex VTP programme within busy referral hospitals in an HIV-endemic region. The study uses a novel ‘non-silo practice score’ as a proxy for assessing related attitudes to determine integrated care. The questionnaire underwent face validity, although no construct validity testing was done. The transferability of the data in this study has to be seen in the context that the province chosen to house this study has the highest HIV antenatal seroprevalence rates in the world and may not represent areas where HIV poses less of a burden.

## Conclusion

This study evaluates in-depth knowledge and attitudes across professions and specialist disciplines in HIV-burdened referral hospitals, and identifies significant gaps in training and care that need review. These gaps are important to address, to ensure SA can eliminate vertical transmission of HIV. The study corroborates the important need for formal training. Still, it strongly advocates that this training be approached regularly from an interprofessional and interdisciplinary framework within referral hospitals. In addition, specific emphasis in training must be placed on identifying acute HIV infections in HIV-negative women and HIV-positive infants in testing strategies. These modifications in the training framework of the VTP programmes may assist in ensuring integrated care and get SA to improve further and possibly eliminate vertical HIV transmission.
